# A novel simplified sonographic approach with fluoroscopy-controlled L5 transforaminal epidural injections in patients with high iliac crest: a retrospective study

**DOI:** 10.1186/s40981-024-00725-0

**Published:** 2024-07-20

**Authors:** Haichang Yang, Hongyan Wang, Jie Lu, Ling Hu

**Affiliations:** 1 Department of Pain Management, The Second Hospital of Tangshan, no. 21 Jianshe North Road, Lubei District, Tangshan City, 063015 Hebei Province China; 2 Department of Pain Management, Xuanwu Hospital, Capital Medical University, No. 45 Changchun Street, Xicheng District, Beijing, 100053 China; 3 Department of Pain Management, Beijing Nuclear Industry Hospital, No. 2 Nan Fourth Lane, Sanlihe Street, Xicheng District, Beijing, 100045 China

**Keywords:** Transforaminal epidural injection, Low back pain, Ultrasound guidance, Fluoroscopy guidance, Numeric rating scale, Modified Oswestry Disability Questionnaire score, Radiation exposure

## Abstract

**Background:**

To explore a novel ultrasound (US) modality for lumbar transforaminal epidural injections (TFEIs) in patients with low back pain (LBP) and L5 radicular pain combined with high iliac crest (HIC).

**Methods:**

One-hundred and forty-one patients were retrospectively stratified into two groups based on the treatment they received: novel group, receiving US-guided and fluoroscopy (FL)-controlled TFEIs using a sagittal oblique approach between the superior articular process of L5 and S1, and control group, receiving US-guided TFEIs with conventional transverse approach combined with FL confirmation. Accuracy of contrast dispersing into lumbar epidural space was set as the primary endpoint. Radiation dosages, procedure time, numeric rating scale (NRS) scores, Modified Oswestry Disability Questionnaire (MODQ) scores, adverse events, and rescue analgesic requirement were also recorded. The generalized liner mixed model (GLMMs) was employed to compare the repeatedly measured variables between groups, taking individual confounding factors as covariance.

**Results:**

The accuracy of TFEIs was 92.8% and 65.2% in novel and control group, with a significant difference of 26.7% (95% *CI*: 15.4%, 39.8%) between two modalities (*p* < 0.001). Significant pain relief was observed in novel group as opposed to control group after one injection. Procedure time in novel group (8.4 ± 1.6 min) was shorter than control group (15.8 ± 3.5 min) (*p* < 0.001) with less radiation dosage (3047 ± 5670 vs. 8808 ± 1039 μGy/m^2^, *p* < 0.001). Significantly, lower incidence of L5 paresthesia occurred in novel group. Statistical differences of NRS scores between the novel and control group were reached at 1 week after procedure (1 (*IQR*: − 1–3) vs. 3 (*IQR*: − 1–7), *p* = 0.006), while not reached at both 1- (1 (*IQR*: 0–2) vs. 1 (*IQR*: − 1–3), *p* = 0.086) or 3-month follow-up (0 (*IQR*: − 1–1) vs. 1 (*IQR*: 0–2), *p* = 0.094). Both groups showed similar functional improvement (*F* = 0.103, *p* = 0.749) during follow-up.

**Conclusions:**

The novel sonographic technique provided superior accuracy needle placement and better pain-relieving effect through one injection as compared to the routine transverse approach. Consequently, in situations where the HIC imposed limitations for TFEIs performance on L5, the novel technique should be recommended to consider increasing accurate puncture, minimizing radiation exposure, consuming procedure time, and reducing the risk of neuraxial injury.

## Introduction

Low back pain (LBP) is an overwhelming health problem, defined as pain, muscle tension, or stiffness localized below the rib margin and above the inferior gluteal folds with or without radiculopathy [1]. The peak prevalence ranges from 28 to 42% in patients between 40 and 69 years [2]. It is usually self-limited to 6 weeks or less, while 10-40% of cases develop chronic pain or frequent relapse if exceeding 12 weeks [3]. Chronic LBP is associated with increased level of pain and severe functional disability, significantly influencing the health-related quality of life (HR-QoL) and accounting for high economical costs [4]. In consequence, it is crucial to take supportive management to improve pain and functional status. According to clinical practice guidelines, for patients failing to the first-line conservative therapies, the evidence is good for invasive treatments including nerve root block and transforaminal epidural steroid injections (TFESIs) [5, 6]. Fluoroscopy (FL) guiding with contrast-enhanced technique has been proved as a routine approach. In unique cases, computed tomography (CT) can be used as the primary approach [7].

Recently, ultrasonography becomes even more popular with its advantages over traditional radiologic method by offering portable, direct visualization, real-time guidance, and radiation-free. To our knowledge, there are a few previous studies reporting the concomitant use of US with FL [8]. But limited studies concern on LBP involving the most common involved fifth lumbar (L5) nerve junction with high iliac crest (HIC), which was derived from Choi’s degree system and defined as the highest point of the iliac crest exceeding the mid of L5 pedicle [9]. The transforaminal access can be very challenging even for experienced doctors because of the obstructive anatomy of HIC.

Based on our clinical experience, a novel approach using a step-by-step sonographic scan, characterized by a funnel-like hyperechoic structure, was firstly described. We hypothesized that the novel approach would facilitate a superior accuracy of needle replacement as opposed to the routine transverse method. Hence, the retrospective study was to compare the effect and facilitation of US-guided TFSIs with FL confirmation between the novel and conventional approach for LBP with L5 radicular pain in patients accompanying with HIC.

## Methods

### Study design and participant selection

The retrospective study was conducted in accordance with the principles of the Declaration of Helsinki and following the Strengthening the Reporting of Observational Studies in Epidemiology (STROBE) guidelines [10]. The ethics was approved by the Ethics Examining Committee of Human Research of Beijing Xuanwu Hospital, Capital Medical University (xw-ky-2023109). Written informed consent was obtained from all participants.

Patients admitted to the Pain Department of Capital Medical University Beijing Xuanwu Hospital and underwent TFSIs for the treatment of lumbar radicular pain between January 1, 2022, and September 30, 2023, were screened through the electronic medical records (EMRs). Inclusion criteria were as follows: (1) diagnosis of chronic LBP according to guideline from the American College of Physicians and the American Pain Society [11] and presenting unilateral radicular pain secondary to a lumbar herniated disk (LHD) or foraminal stenosis resulting in symptoms of L5 radiculopathy, (2) confirmation of a LHD or foraminal stenosis at the ipsilateral L5 level by CT or magnetic resonance imaging (MRI), (3) clinical symptoms did not respond to commonly used conservative treatments for at least 6 weeks [12], and (4) ipsilateral iliac crest exceeding the midpoint of the L5 pedicle based on the lateral radiography [0]. Patients were excluded if they had BMI ≥ 30 kg/m^2^, a history of surgery on the lumbar spine, chronic psychiatric illnesses, convention to other invasive treatments, and incomplete medical data or lost to follow-up.

**Fig. 1:**
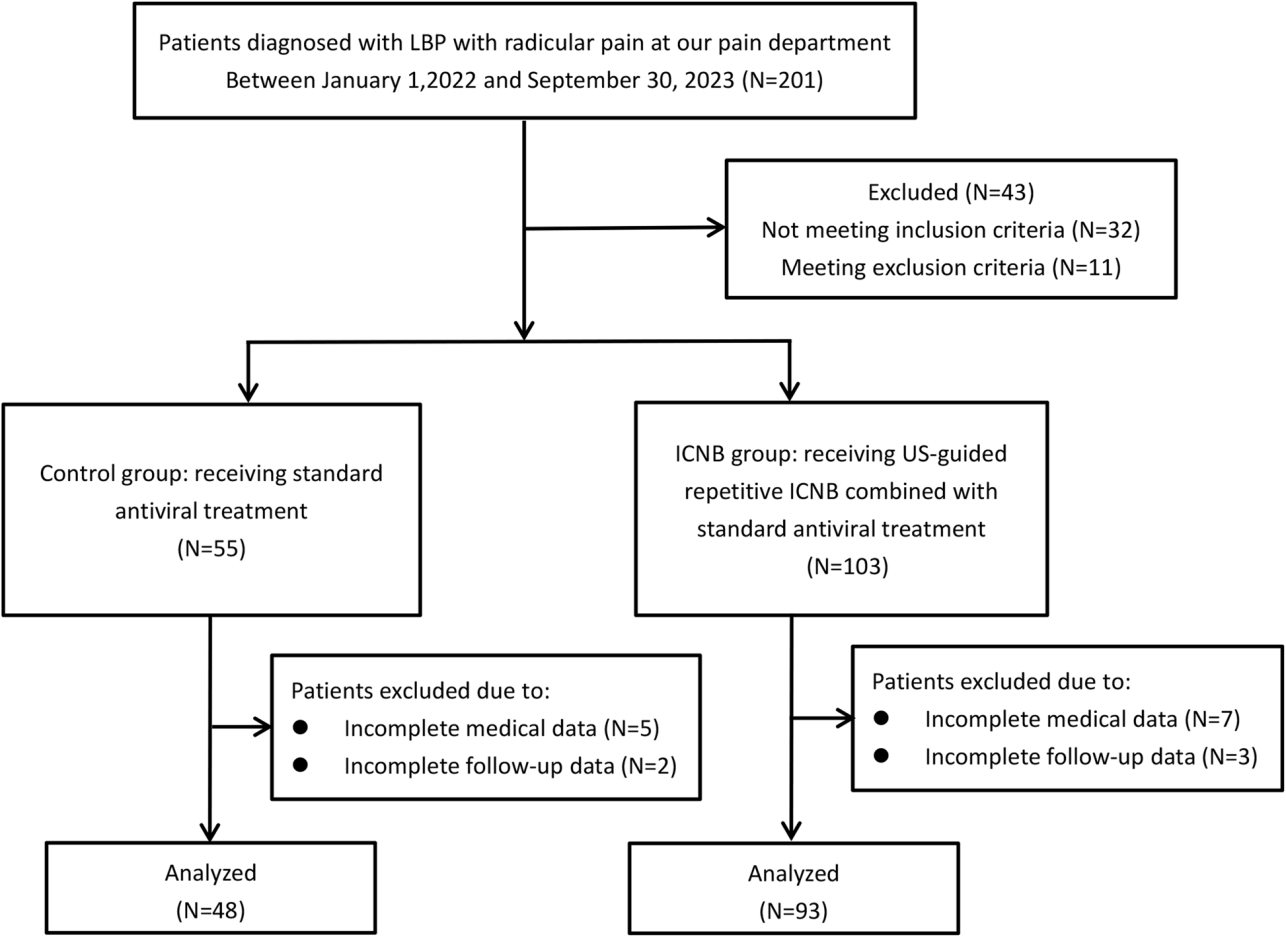
The flow chart of the study

A total of 141 consecutive patients were divided into two groups based on the approach used during US guidance: the novel group, receiving US-guided PRF on the L5 nerve root using the novel approach and verified by FL, or the control group, receiving the same treatment using the transverse method during US guidance (Fig. 1).

### Procedure management

The procedures were performed by the same team of pain physicians, who were with more than 5 years’ expertise in interventional techniques for chronic spinal pain. All patients were prepared in a prone position with a pillow under the lower abdomen to compensate for the lumbar lordosis in the sterile operating room and monitored by electrocardiography (ECG), noninvasive blood pressure, and pulse oximetry (SpO_2_).

### Novel group

**Fig. 2:**
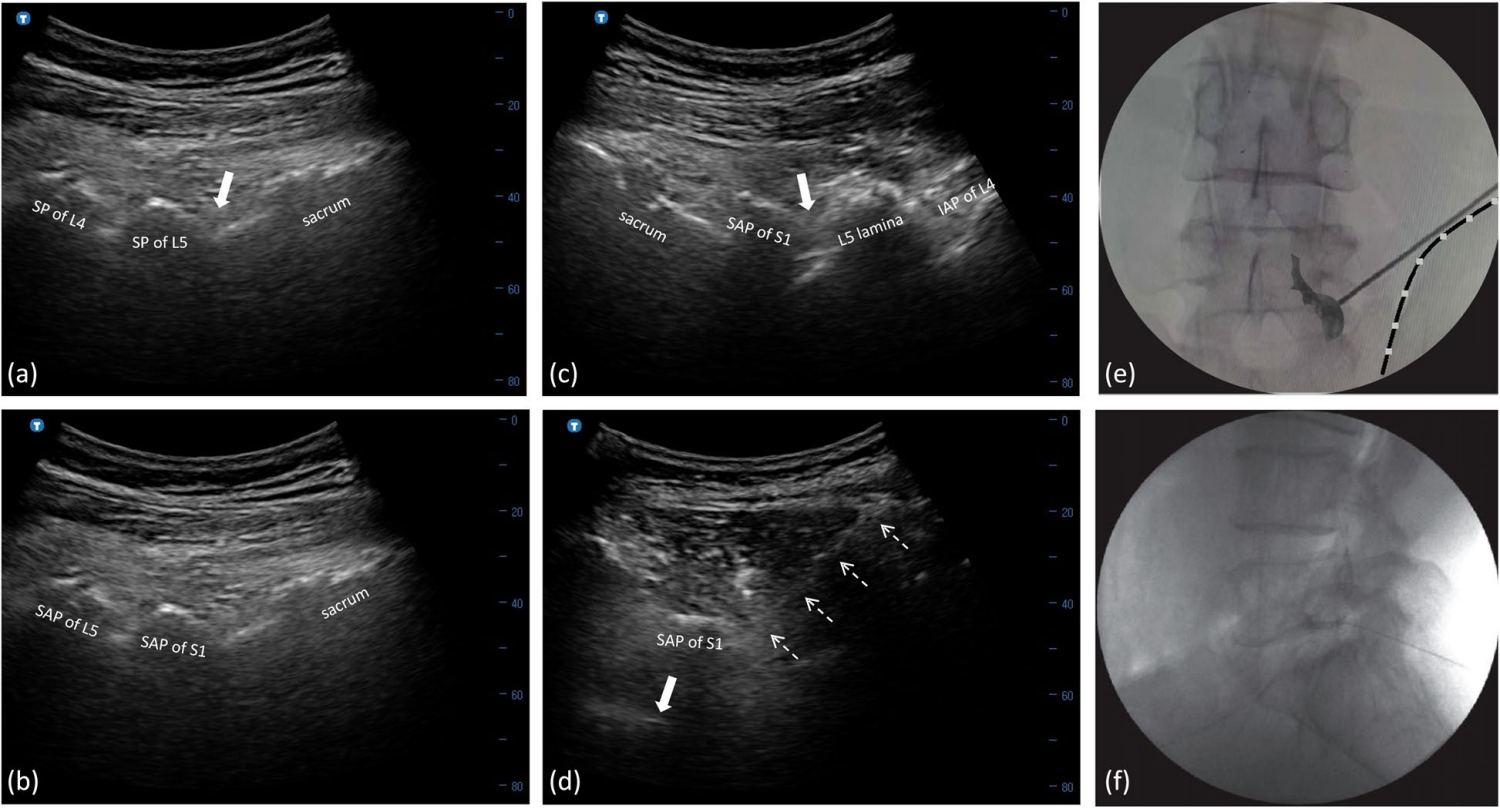
**a** Ultrasound pictures showing that a funnel-shaped hyperechoic structure (solid arrow) was consequently identified between the spinous process (SP) of L4 and L5 and the sacrum on the paramedian longitudinal scans. **b** The facet joint of L5–S1 appeared as a hyperechoic line known as the “camel’s hump” until the funnel-shaped hyperechoic structure completely disappeared. **c** The lateral position of the transducer at the inside border of S1-SAP (superior articular process). **d** By rotating the transducer about 45–60° from the final longitudinal scanning towards the ipsilateral iliac crest with a successional visualization of the unique funnel-like hyperechoic signal lying at the lower inside of S1-SAP, the needle (open arrow) was advanced to the targeted L5 foramen under the uninterrupted hyperechoic bony line of the S1-SAP using an in-plane technique. **e** and **f** Fluoroscopic anteroposterior and lateral view verified an intraforaminal contrast dispersion in patients with high iliac crest following the ultrasound-guided novel approach

After sterilization, a 2–5-MHz curvilinear probe (Labat, Shenzhen Huasheng Medical Technology Co., Ltd., China) was longitudinally positioned on the midline to visualize the targeted segment of L5 spine. A funnel-shaped hyperechoic structure posterior to the dorsal dura was consequently identified between the L5 spinous process (SP) and the S1 sacral crest. By moving the probe further towards the lateral, the hyperechoic funnel situated between the L5 lamina and the sacrum gradually became smaller in sequence (Fig. 2a). Until it completely disappeared, the facet joint of L5–S1 appeared as a hyperechoic line known as the “camel’s hump” with its acoustic shadowing beneath on the sonographic long-axis view (Fig. 2b). Subsequently, the probe was moved to the opposite direction targeting at the inside border of superior articular process (SAP) of S1 (Fig. 2c). After completion of the longitudinal scanning, the probe was then turned about 45° to the ipsilateral iliac crest to gain an oblique line, taking a successional visualization of the unique funnel-like hyperechoic signal lying at the lower inside margin of the S1-SAP as a reference point. In this oblique view, typically, the posterior side of the targeted L5 foramen was identified under the uninterrupted hyperechoic bony line of the S1-SAP (Fig. 2d). Color Doppler mode was manipulated to avoid the critical vessels including radicular or segmental vessels adjacent the puncture path [13]. The needle (Nerve Block Needle, 22G, 15 cm, Shenzhen Huasheng Medical Technology Co., Ltd., China) was advanced towards the outer edge of the targeted hyperechoic line and gently slipped beside it until the loss of resistance (LOR) was achieved using an in-plane approach. The precise position of needle tip was adjusted by advanced a little deeper and not beyond the 6 o’clock position of the vertebral pedicle under FL without contrast medium confirmation. Upon negative aspiration, contrast spread pattern was then verified followed by further injection of 1 ml of contrast medium. A test injection was administered with 0.5 ml of 1% lidocaine. A total of 2 ml of mixture including 0.5 ml of 2% lidocaine (20 mg/ml) and 1 ml of betamethasone (5 mg:2 mg/ml) was subsequently injected.

### Control group

After identifying the L5 spinal level in the same sagittal US image as the novel group, the curvilinear probe was placed perpendicular to the long axis to view the SP, lamina, facet joints, and transverse process (TP) in sequence. The probe was slightly moved upward or downward to detect the root portions of TP, in which image the posterior edge of the L5 neural foramen was delineated as a hyperechoic bone structure with its underlying acoustic shadow. After confirmation of no critical vessels adjacent the peri-radicular area by color Doppler mode, the same needle as the novel group was positioned from the lateral to the middle using the transverse axis in-plane approach under the real-time US guidance. The precise position of needle tip was then adjusted and confirmed under FL. If the L5 neural foramen was difficult to reach due to the HIC, the procedure might be terminated by administering an agent around the nerve root. The contrast-spread distribution was also verified following the negative aspiration. After the same test injection was confirmed fluoroscopically, the same mixture as the novel group was injected.

### Outcomes measurement

All data were collected through the EMRs. Pain severity was assessed by the numerical rating scale (NRS) ranging from 0 (no pain) to 10 (worst pain). Successful treatment response was defined as pain relieving ≥ 50% reduction from baseline NRS score at 1 week after the initial injection on the basis of clinical consensus. Patients reported < 50% reduction in pain relief received the second injection following the same protocol as the first one. Modified Oswestry Disability Questionnaire (MODQ) scores were employed to measure the LBP-related functional disability, where higher scores indicated greater limitation. It consists of 10 items addressing personal care, lifting, walking, sitting, standing, sleeping, social life, traveling, and employment/homemaking. Each item is scored from 0 (no disability) to 5 (maximal disability) [14]. Contrast media dispersion patterns were judged by FL, which were graded as follows: (1) “intra-muscular” type when contrast accumulated locally in muscles without peri-radicular filling, (2) “peri-radicular” type when contrast spread only along the nerve root but not reaching the articular pillars, and (3) “epidural” type if contrast spread exceeding the outer margin of the articular pillars. Procedure time was defined as starting from the ultrasound probe touching the skin of the effective area to the end of the injection. Number of needle insertions and attempts until contrast was given, and radiation dosages, complications, and rescue analgesia were also recorded.

The primary outcome was the accuracy of needle tip placement, as confirmed by the contrast-spread pattern of “epidural type.”

### Sample size calculation

Sample size was calculated using PASS software version 16 (NCDS, LLC, Kaysville, UT, USA) before patients’ screen for eligibility. Because of no certain evidence about the accuracy of TFSI at L5 level using transverse approach under US guidance in patients with high iliac crest, the sample size calculation was based on consensus after a series of expert discussion meetings estimating a 75% accuracy rate. We would like to decide that the novel approach would be adopt if it has an accuracy rate of at least 85%. Using the one-sided Farrington and Manning likelihood score test to reach a power of 90% at group sample size ratio of 2, we came up with 44 patients in control group and 88 cases in novel group, when the difference of the actual accuracy rate between two approaches ranged from 20 to 25%.

### Statistical analysis

Statistics were performed using a SPSS 22.0 (SPSS Inc, Chicago, IL, USA). Significance was accepted at *p* < 0.05. Kolmogorov-Smirnov *Z*-test was performed for the normal distribution of all data. Categorical data, normal distribution data, and non-normally distributed data were expressed as frequencies/percentages, mean ± standard deviation (SD), and median ± interquartile range (IQR) and compared using the chi-squared test, Student’s *t*-test, and Mann-Whitney *U*-test between groups. Changes in NRS scores and MODQ scores were assessed using the generalized liner mixed model (GLMMs) taking age, gender, BMI, and iliac height as covariance. Paired and independent *t*-test derived from GLMMs for repeated measurements were applied for within-group and between-group comparisons, respectively.

## Results

Figure 1 illustrated the profile of the trial. Patients’ demographic and clinical characteristics were well balanced between the two groups before treatment (Table 1).

**Table 1:** Patients’ demographic and clinical characteristics at baseline

**Variables**	**Novel group (*N* = 93)**	**Control group (*N* = 48)**	***p***
Age (years) (mean ± SD)	65.07 ± 7.51	66.03 ± 8.29	0.640
Female sex, *n* (%)	53 (57.0%)	25 (52.1%)	0.596
BMI	24.58 ± 3.12	23.62 ± 2.53	0.957
Iliac height (mm)	36.56 ± 4.84	37.61 ± 5.68	0.712
Affected side, *n* (%)			0.375
*Left*	44 (47.3%)	27 (56.3%)	
*Right*	49 (52.7%)	21 (43.8%)	
Diagnosis, *n* (%)			0.449
*Lumbar disc herniation*	66 (71.0%)	31 (64.6%)	
*Lumbar foraminal stenosis*	27 (29.0%)	17 (35.4%)	
Type of pain			0.858
*L5 radicular pain without LBP*	39 (41.9%)	20 (39.6%)	
*L5 radicular pain with LBP*	54 (58.1%)	28 (60.4%)	
Duration of pain (months) (mean ± SD)	10.86 ± 3.53	10.51 ± 3.86	0.436
NRS scores at baseline (median ± IQR)	7 (5, 10)	6 (4, 8)	0.698

*NRS* numeric rating scale, *BMI* body mass index, *LBP* low back pain, *SD* standard deviation, *IQR* interquartile range

As shown in Table 2, the accurate rate of epidural contrast dispersion at the L5 level occurred in 92.8% of cases in novel group. It was statistically superior to the control group (65.2%), and the mean difference equaled 26.7% (95% *CI*: 15.4%, 39.8%) (*p* < 0.001). As opposed to control group, the procedure time in novel group was significantly shorter (8.4 ± 1.6 min vs. 15.8 ± 3.5, *p* < 0.001). The number of needle attempts and insertions until contrast was given was significantly less in the novel group when compared to the control group (1 (*IQR*: 0–2) (range: 1–3) vs. 5 (*IQR*: 3–7) (range: 1–7), *p* < 0.001). Additionally, the radiation dosage in the novel group was lower than that in the control group (3047 ± 570 vs. 8708 ± 1039 μGy/m^2^, *p* < 0.001).

**Table 2:** Comparison of procedure variables for two groups

**Outcomes**	**Novel group (*N* = 93)**	**Control group (*N* = 48)**	**Difference in rate (95% *CI*)**	**Rate ratio (95% *CI*)**	***χ***^**2**^**/t/Z value**	***p***
**Types of injections**					9.416	0.003
*One injection*	75 (80.6%)	27 (56.3%)	24.4% (8.2%, 40.6%)	3.241 (1.503, 6.985)		
*Two injections*	18 (19.4%)	21 (43.8%)				
**Contrast spread pattern**	***N* = 111**	***N* = 69**			27. 930	< 0.001
*Epidural dispersion*	103 (92.8%)	45 (65.2%)	27.6% (15.4%, 39.8%)	1.423 (0.897, 2.257)		
*Peri-radicular dispersion*	7 (6.3%)	14 (20.3%)				
*Intramuscular dispersion*	1 (0.9%)	10 (14.5%)				
**Number of needle insertions until contrast given (median ± IQR)**	1 (0, 2)	5 (3, 7)			− 6.312	< 0.001
**Procedure time (min) (mean ± SD)**	8.39 ± 1.63	15.83 ± 3.46			− 10.653	< 0.001
**Radiation dosage (μGy m**^**2**^**) (mean ± SD)**	2203.56 ± 898.55	8707.50 ± 1021.10			− 14.734	< 0.001

*IQR* interquartile range, *SD* standard deviation

**Fig. 3:**
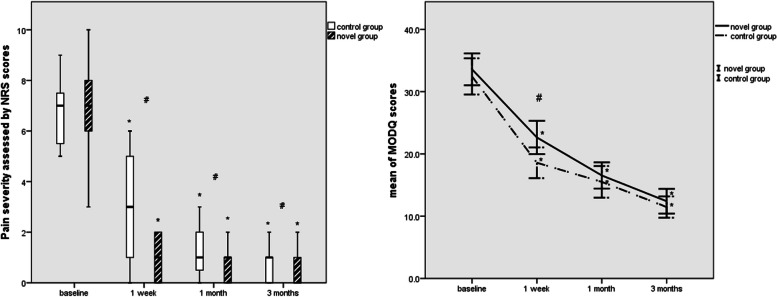
**a** Boxplot of numerical rating scale (NRS) scores for pain intensity at baseline, 1-week, 1-month, and 3-month follow-up after the first injection. **b** Changes in pre-TFEIs and post-TFEIs Modified Oswestry Disability Questionnaire scores. #*p* < 0.05 compared between group, *an adjusted *p* < 0.017 compared within group. TFEIs, transforaminal epidural injection

Successful pain relief (≥ 50% reduction in NRS scores) after the first TESI was noted in 80.6% of patients in novel group at up to 1 week, and in 56.3% of cases in control group, which illustrated that significantly more patients received the second injection in control group if compared to novel group (*p* = 0.003). Figure 3 revealed the changes of NRS scores and MODQ scores from baseline to 1 week, 1 month, and 3 months after procedure in both groups. According to GLMMs analysis, significantly greater improvements were observed in novel group as opposed to control group with regard to NRS scores (*F* = 7.149, *p* = 0.008). Between-group analysis showed that lower means of NRS scores were observed in novel group as compared to control group at all time points during follow-up; nevertheless, the statistical differences were reached at 1 week (1 (*IQR*: − 1–3) (range: 0–3) vs. 3 (*IQR*: − 1–7) (range: 0–7), *p* = 0.006), while not reached at both 1 (1 (*IQR*: 0–2) (range: 1–2) vs. 1 (*IQR*: − 1–3) (range: 0–3), *p* = 0.086) and 3 months (0 (*IQR*: − 1–1) (range: 0–2) vs. 1 (*IQR*: 0–2) (range: 0–2), *p* = 0.094). Based on the GLMMs analysis, the MODQ scores also showed a significant decrease from baseline to 1 and 3 months after procedure in both two groups. Nevertheless, no significant difference was observed between two groups during the whole 3-month follow-up period (21.29 ± 1.22 vs. 19.50 ± 1.22, *F* = 1.075, and *p* = 0.300). Significant difference in MODQ scores was observed between the two groups only at post-1-week follow-up (18.75 ± 11.17 vs. 22.64 ± 12.17, *p* = 0.028), whereas not observed at post 1 month (15.51 ± 11.49 vs. 16.53 ± 9.61, *p* = 0.537) and 3 months (11.47 ± 7.74 vs. 12.40 ± 9.04, *p* = 0.481). In the usage of rescue analgesia during follow-up, no significant difference was observed between the two groups (9.0% vs. 16.7%, *p* = 0.179).

Serious complications including spinal infarction, visible hematoma, and motor deficit were not encountered in both two groups. Only 6.3% of nerve root irritation with the presence of transient radiating pain or paresthesia occurred in novel group, however 27.5% in control group (*p* < 0.001). Intravascular contrast spread was not observed in any patients. Complications related to transforaminal steroid and local anesthetics including dizziness, nausea, vomiting, and facial flushing were detected in 6.3% and 8.7% of cases in novel and control group (*p* = 0.565), which were all resolved within 30 min.

## Discussion

To date, there is no accepted standard approach under US guidance for the treatment of LBP and L5 radicular pain. The present study was firstly conducted to estimate the accuracy, clinical outcomes, and benefits of TFSIs on L5 in patients with HIC, supporting the use of a simplified novel approach of sonographic guidance technique with conventional FL verification.

The pathophysiology of LDH and/or foraminal stenosis usually involves both mechanical compression and chemical sensitization, which may cause the development of epidural inflammation and stimulate the spinal nerve roots, leading to the edema and the increasing vascular permeability of nerve roots, and then causes LBP and radicular pain [12]. Therefore, peri-radicular infiltration steroid was proved to be useful in alleviating the chemical irritation of nerve roots and hence radicular pain because it inhibited the synthesis of various pro-inflammatory mediators and suppressed neural transmission within the nociceptive C-fibers [15]. Systemic review and meta-analysis revealed that the transforaminal approach allowed the true ventral needle placement in epidural space and consequently had the best potential of the ESI techniques to reduce pain and improve function in managing LBP and radicular pain, indicating levels II-1 or II-2 evidence, with a 1C/strong recommendation, which was similar as the American College of Occupational and Environmental Medicine (ACOEM) guidelines [13, 14, 16].

Although FL guidance has been proved to be a clinical efficacious modality for lumbar TFEIs, this technique is seen as likely to expose operators and patients to radiation hazards [17]. Over the past decade, US-guided techniques provide better visualization of soft tissue, peripheral nerve, and vascular structure over the routine FL-guided procedure. Needle placement can be tracked in real-time image guidance while avoiding exposure to radiation [18]. Recently, the transverse US scanning has been proved to be helpful in viewing the sonographic anatomy of para-radicular area and/or neural foramen in the lumber spine, which allows the placement of needle to the sub-pedicular area using in-plane approach [19, 20]. Compared with fluoroscopic technique alone, in which the presence of contrast within the intravertebral foramen only occurred in 18.7% of cases, the accuracy of needle tip placement was reported as up to 85% for low back and radicular pain when a transverse view of US with subsequently FL validation was employed: 100% in L3–4 level while 80% in L4–5 level by previous randomized controlled trials (RCTs) [21, 22]. Nevertheless, transforaminal approach is more difficult and challenging at the L5 level in patients with the presence of some limiting factors including thickened transverse processes, facet joints morphology and HIC, and sometimes even at L4 level [23]. The HIC and the inclination of the L5–S1 level frequently obstruct the transforaminal approach, resulting in a steeper trajectory angle [24].

Some researcher advocated a longitudinal US scan technique using the respective intertransverse ligament between two adjacent TPs as the landmark. However, the intra-foraminal contrast-spread pattern was detected in only 20–30.2% of cases. Besides, needle placements at the L5 level failed in 15.2% of patients, because the prominent ilium inhibited the needle from passing, especially when the in-plane technique was employed [25]. Based on our successful clinical experience, the extra-foraminal area closely next to the SAP of S1 level was firstly set as the targeted landmark to overcome the obstruction of HIC in the present study. The US probe was subsequently rotated obliquely after reaching the abovementioned endpoint. Therefore, the needle tip was placed deeper and more medially to reach at the posterior area of L5 foramen. According to our results, the present novel sonographic modality revealed favorable results, and a significantly higher rate of intra-foraminal dye spread was found in novel group as opposed to control group. Furthermore, the novel approach not only significantly increased the accuracy rate of contrast dispersing into L5 neural foramen but also significantly shorter the procedure time, both of which was similar to the conventional transverse approach used in patients without HIC reported in previous study (accuracy of LTFEI was 90.2%, and procedure time was 8.87 ± 1.12 in US group) [20]. This might be on account for facilitating needle placement with significantly less numbers of needle attempts and insertions to overcome the obstructive anatomy to transforaminal access until the contrast given. In addition, the more precise visualization for needle entry also decreased the numbers of FL images for adjusting the placement of needle tip, resulting in lower radiation dosage in novel group, which was also comparable to the US-guided TFEIs using the routine transvers method in cases without HIC [22].

With regard to complications, a comparable incidence of minor side effects associated with TFEIs was reported in both two groups, which was consistent with previous literature review reporting 2.4 to 9.6% [22]. Although major complications in lumbar TESIs were extremely rare, of those that did report, spinal cord injuries from needle placement, spinal cord infarct, and epidural hematoma and infections were documented as catastrophic case reports, but most of them could be avoided by accurate needle placement under image-guided injections and sterile techniques [26, 27]. As the transverse technique was based on lamina visualization, the intraspinal structure was totally shadowed by the hyperechoic bony structure of the lumbar lamina. Therefore, appropriate measures, e.g., the depth of needle insertion, should be strictly emphasized as further progress might result in needle progress into the neuraxial compartment without notice and cause the permanent damage [28]. Based on the novel technique using the oblique view, the needle tip was approached by the in-plane technique, enabling the real-time US visualization of the entire needle path. Notably, the unique funnel-like hyperechoic sign representing the neuraxial content is located far away medial inferior the targeted area. Therefore, it was unlikely to stab the corresponding L5 nerve root and injury the spinal cord. According to our results, a paresthesia of the nerve due to the stab with the needle tip occurred only in 7 patients (6.3%) in novel group, while it significantly increased to 27.5% in control group.

Besides this, significantly, more patients in novel group experienced a successful pain relief at 1 week after the first injection, using ≥ 50% reduction in NRS scores, as opposed to control group (*p* = 0.003), and reaching the previous reported overall success rate around 76–88% [29]. In general, NRS scores and MODQ scores were significantly improved at 1- and 3-month follow-up in both two groups as compared to their baseline values, which was consistent with the systematic review to assess the efficacy of TE-ESI in patients with LBP and unilateral lumbosacral radicular pain [30, 31]. It must be pointed out that the intergroup difference in the improvement of both NRS scores and MODQ scores at 1 and 3 months after the injections was not statistically significant, respectively. To our knowledge, although poor accuracy via the conventional transverse approach which translated to imprecise needle placement to L5 foramen directly results in inferior pain reduction and functional improvement than novel group, repeating the injection might compensate for the disappointing clinical outcomes.

There were several limitations. Firstly, undetected confounders and possible bias might occur on account of the nature of retrospective analysis with observational data. Secondly, the performance of US guidance technique highly depended on the experience of the operator. Thirdly, similar pain relief and functional improvement were observed in both two groups during 1- and 3-month follow-up, which might be owing to the limited sample size. Fourthly, confounders might occur in the efficacy analysis, because parts of patients used rescue analgesics after TFEIs. In the future, a well-designed, randomized, controlled study was needed to validate our results.

## Conclusion

In cases with LBP and L5 radicular pain treated by TFEIs, the novel US modality is a feasible and effective technique that allowed to overcome the limitations imposed by the HIC. Compared to the conventional transverse approach, it showed advantages in terms of increasing needle placement accuracy, decreasing the risk of neuraxial injury, attenuating radiation exposure, reducing performance time, and enhancing post one-injection clinical outcomes.

## Data Availability

The data and materials that support the findings of this study are available from the corresponding author, Professor Ling Hu, upon reasonable request.

## Abbreviations

LBP: Low back pain
TFEI: Transforaminal epidural injection
US: Ultrasound
FL: Fluoroscopy
CT: Computed tomography
MRI: Magnetic resonance imaging
HIC: High iliac crest
NRS: Numeric rating scale
MODQ: Modified Oswestry Disability Questionnaire
HR-QoL: Health-related quality of life
LHD: Lumbar herniated disk
AP: Anteroposterior
TF: Transforaminal
SAP: Superior articular processes
ESI: Epidural steroid injections
EMRs: Electronic medical records
ECG: Electrocardiography
SpO2: Pulse oximetry
IQR: Interquartile range
CI: Confidence interval
RCTs: Randomized controlled trials
